# Developing and maintaining a nose-to-brain map of odorant identity

**DOI:** 10.1098/rsob.220053

**Published:** 2022-06-29

**Authors:** Ana Dorrego-Rivas, Matthew S. Grubb

**Affiliations:** Centre for Developmental Neurobiology, Institute of Psychiatry, Psychology and Neuroscience, King's College London, London SE1 1UL, UK

**Keywords:** olfaction, olfactory sensory neurons, odorant receptors, olfactory bulb, axon guidance, development

## Abstract

Olfactory sensory neurons (OSNs) in the olfactory epithelium of the nose transduce chemical odorant stimuli into electrical signals. These signals are then sent to the OSNs' target structure in the brain, the main olfactory bulb (OB), which performs the initial stages of sensory processing in olfaction. The projection of OSNs to the OB is highly organized in a chemospatial map, whereby axon terminals from OSNs expressing the same odorant receptor (OR) coalesce into individual spherical structures known as glomeruli. This nose-to-brain map of odorant identity is built from late embryonic development to early postnatal life, through a complex combination of genetically encoded, OR-dependent and activity-dependent mechanisms. It must then be actively maintained throughout adulthood as OSNs experience turnover due to external insult and ongoing neurogenesis. Our review describes and discusses these two distinct and crucial processes in olfaction, focusing on the known mechanisms that first establish and then maintain chemospatial order in the mammalian OSN-to-OB projection.

## Graphical abstract

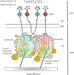

## 1. Introduction

Mammalian olfaction is a critical sense for odour identification, discrimination and memory, and ultimately for survival. Odour detection starts in the olfactory epithelium (OE) of the nose, with sensory transduction performed by olfactory sensory neurons (OSNs). OSNs have an apical dendrite with numerous cilia expressing odorant receptor (OR) molecules, which, by binding specific odorant molecules, trigger an intracellular transduction cascade that leads to action potential generation and propagation along OSN axons. Each individual mature OSN expresses just one allele of one OR, selecting from a family of over 1100 OR genes [1–5]. OSNs expressing the same OR, known as ‘like-OSNs’, are not spatially clustered in the OE but are instead distributed in salt-and-pepper, interspersed patterns within a range of complex and overlapping nasal expression zones [6–9]. While some broad spatial organization is present in the way that these expression zones send projections to central targets (see §3), the most striking feature of the nose-to-brain map is the extreme local precision of like-OSN axonal targeting.

**Figure 1.**
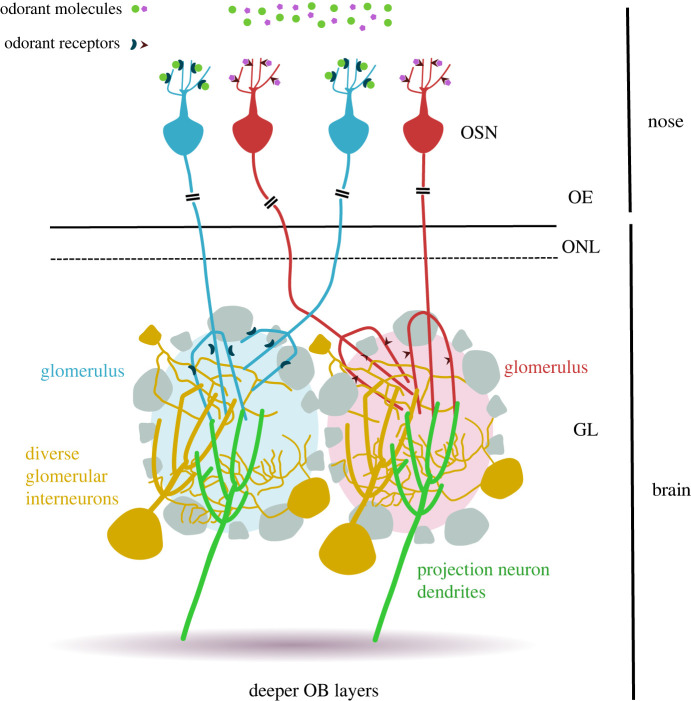
Mature organization in the projection of olfactory sensory neurons to the olfactory bulb. In the olfactory epithelium (OE), different odorants bind to odorant receptors present on the cilia of olfactory sensory neurons (OSNs). These receptors can also be found on OSN axon terminals. OSNs project long axons that form the olfactory nerve, and then the olfactory nerve layer (ONL) in the olfactory bulb (OB) in the brain. They terminate in glomeruli in the OB's glomerular layer (GL), where they form synapses with the dendrites of diverse glomerular interneurons and OB projection neurons. OSNs expressing the same odorant receptor target the same glomerulus, represented in the figure by the blue/red colours.

OSNs project their axons to the main olfactory bulb (OB), the first region of the brain involved in processing olfactory information. OSN axons terminate in spherical structures known as glomeruli. These are situated in the aptly named glomerular layer of the OB, and consist of largely soma-free axonal and dendritic neuropil surrounded by the cell bodies of bulbar interneurons and projection cells (figure 1). Each mature glomerulus is targeted by the axons of just one class of like-OSNs [10,11] (figure 1), and each population of like-OSNs sends axons to terminate in usually a single glomerulus or, at most, a few glomeruli in both the medial and lateral halves of each OB [6,12,13]. With 1141 identified mouse OR genes at the latest estimate [5] and approximately 3600 glomeruli in each mouse OB [14], there are an average of approximately three glomeruli targeted by each population of like-OSNs per OB [8], and these are distributed across a double map with around one or two like-OSN-targeted glomeruli in each medial or lateral half-bulb. The relative positions of individual glomeruli are reasonably symmetrical between the left and right OBs of individual animals, and are also similar, but not identical, between different animals [15–17]. Because the specific OR expressed by a population of like-OSNs determines not only their selectivity to odorant stimuli but also their axonal coalescence in specific regions of the OB, this anatomical organization produces a consistent chemospatial map in the brain, in which the identity of individual odorants is encoded at least in part by the spatial patterns of glomeruli they activate [15,18–20]. How does such a precise and complex map form during development? And how is it maintained throughout adult life? Our review addresses precisely these questions, dealing exclusively—for reasons of focus—with the establishment and maintenance of the main olfactory projection in mammals (mice and rats, unless otherwise stated). For excellent coverage of glomerular targeting in other animal models [21–24], the establishment of other olfactory pathways [25,26] or the development of downstream OB circuitry [27], we refer the curious reader to other recent articles on these topics, cited above. Those wishing to obtain diverse and comprehensive treatment of nose-to-brain map development are also directed to some of the many high-quality existing reviews on this topic [28–32].

## 2. Fundamental developmental processes of OSN-to-OB inputs

**Figure 2.**
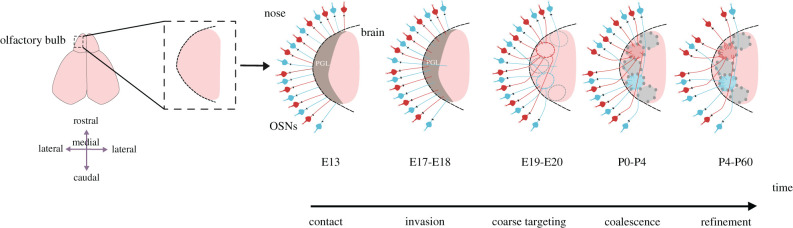
Development of OSN projections to the olfactory bulb. For simplification, only a portion of one olfactory bulb is shown as an example. This is a representation of local-level targeting only; information on coarse positional targeting can be found in §3. OSN axons initially contact the presumptive glomerular layer (PGL) of the olfactory bulb at around embryonic day (E) 13. A few days later, by E17–E18, axons start invading the PGL, with rare exceptions erroneously growing through and past this layer. By E19–E20, OSN axons start to approach the approximate future position of their target glomeruli (shown in dotted outline), with some targeting errors. Axons overshooting the glomerular layer are still present. During the first postnatal (P) days (P0–P4), axons start to coalesce and branch in appropriate glomeruli, which are now delineated by the somas of surrounding glomerular layer interneurons (grey). Some targeting errors persist, while neurons with overshooting axons are eliminated. Later (P4–60), OSNs elaborate their axon terminals in appropriate glomeruli, while erroneous projections are removed.

OSN axons reach the border of the presumptive OB very early in rodent development, around embryonic day (E)13, but they then stall there for a few days before invading the OB's outer layer, forming a rather uniform band of axonal projections at ∼E17-18 (figure 2) [33–36]. The depth of this projection into the OB is extremely tightly controlled—only a few growing OSN axons overshoot the presumptive glomerular layer, and these erroneously deep axons disappear by postnatal day 5 (∼P5) [37] (figure 2). However, although manipulations of neuropilin2-Sema3F signalling [38–40] or olfactory marker protein (OMP; [41]) can cause the persistence of some overshooting OSN axons, it is entirely unclear what the major stop signal is for OSN growth within the presumptive OB. The apical dendrites of bulbar projection neurons (see below) have been suggested as a possible source of this signal [35], but cannot be solely responsible since spatially restricted glomeruli are formed normally in mice which lack these cells entirely [42].

Within the presumptive glomerular layer, the process of OSN axon coalescence into discrete glomeruli begins with the formation of focal axon arbour densities known as protoglomeruli in late embryonic development (∼E19–20 [34–36] figure 2). In early postnatal development further OSN axon segregation and coalescence occurs, together with the migration of surrounding glomerular cell bodies, such that grossly mature, discrete glomeruli can be seen from ∼postnatal day (P)0-4 (figure 2) [36]. Glomerular formation occurs with a pronounced anterior-posterior gradient across the OB, with anterior development usually around 2–3 days ahead of posterior events [36].

Compared to these overall maturation patterns, more precise information about the development of individual glomeruli can be obtained using genetic strategies for selectively labelling populations of like-OSNs [13]. Disappointingly, in mammals these have not yet been coupled with techniques for longitudinal *in vivo* imaging [43], which would enable the dynamics of glomerular formation to be visualized live. However, time series of fixed samples have shown that like-OSN axons begin as a broadly distributed ‘tangle’ over a large surface of the OB. This then organizes into a more localized protoglomerulus, and finally a mature glomerular coalescence, at rates and times that depend upon the OR-expressing population being studied [44,45]. Labelling of two closely related like-OSN populations reveals considerable early overlap in their coalesced projections, followed by later local segregation into individual glomeruli [33,46].

After this initial glomerular segregation, a further refinement process occurs (figure 2). Initial projections of like-OSNs can target multiple glomeruli in each half-OB, often with just a few sparse axons. These weaker, erroneous projections are removed in later postnatal development to leave the mature pattern of approximately 1-2 like-OSN-targeted glomeruli per half-bulb [11,45]. Again, the exact timing of this refinement depends on the OR-expressing population – the P2 glomerulus is almost fully refined in the first postnatal week [45], but the M72 and M71 glomeruli are not completely mature until P20 and P60, respectively [11]. The many-to-one refinement glomerular process does not occur within individual OSN axons, because sparse anatomical label has never identified an individual axon branching within more than one glomerular structure. Postnatally, at least, they are always confined to a single glomerulus, and indeed always to a restricted sub-area of any glomerulus [47,48]. However, maturation of OSN axon branching within glomeruli does occur after birth, with increasing arbour complexity up to around P7 followed by stable maintenance [47] or pruning [48] depending on the timing and/or precise method used to obtain sparse fluorescent labelling. Again, live *in vivo* imaging of individual OSN arbour dynamics [49] and/or OSN sensory response properties [50] would have much to reveal in this crucial postnatal period.

## 3. The development of coarse topography in OSN inputs to the OB

If glomeruli are consistently positioned within the three-dimensional structure of each OB, what developmental mechanisms produce such spatial patterning? In the mediolateral and dorsoventral axes this is achieved with coarse point-to-point topographic mapping. The anteroposterior axis of the OB, however, is established according to unique mechanisms that are dependent upon OR expression.

### 3.1. Mediolateral mapping

The duplication of OSN-to-OB targeting into two distinct glomerular maps per bulb occurs largely around the mediolateral axis, yet despite this being a fundamental anatomical feature of the early mammalian olfactory system, very little is known about its functional importance or its development. We do know that there is coarse overall topography in the nose-to-brain projection such that OSNs in the medial OE generally project to the medial OB, and OSNs in the lateral OE generally project to the lateral OB [51]. We also have a good understanding of the role of one molecule in the maturation of this axis: IGF1, which is expressed in a high-lateral to low-medial gradient in the developing OB [52]. Knocking out its receptor IGF1R—which is normally expressed by OSNs—or deleting both IGF1 and IGF2, leads to a stark lack of lateral glomeruli [52]. IGF1, which can attract OSN axon growth *in vitro*, may therefore be required to bring lateral OSN axons to the lateral OB [52].

### 3.2. Dorsoventral mapping

The dorsoventral axis of the OB is also patterned with canonical axon guidance mechanisms, though with an important temporal component. It also maps like-to-like from nose to brain, with dorsal OSNs projecting to the dorsal bulb and ventral OSNs projecting to the ventral bulb [7,29,53,54]. A key distinguishing feature of this axis is the clear segregation in OSN expression of particular molecules: dorsomedially projecting axons express the enzyme NQO1 [54], while the cell adhesion molecule OCAM is found in axons that project ventrolaterally [53,55]. Dorsally localized OSNs develop earlier than their ventral counterparts, express high levels of Robo2, and are driven dorsally away from the ventral OB by repellent interactions with locally expressed Slit1 and Slit3 [56,57], with a potential additional contribution from Robo1 expressed by olfactory ensheathing glia [58]. Dorsal OSNs also express high levels of Sema3F, and in OSN-specific Sema3F knockout mice there are aberrant dorsal projections of ventral OSNs that highly express the Sema3F receptor Nrp2 [59]. Gain- and loss-of-function manipulations of Nrp2 shift glomerular positions ventrally or dorsally, respectively [59]. The model here is that early arriving dorsal OSN axons secrete Sema3F in the dorsal OB, which then repels Nrp2-expressing, later-arriving ventral OSN axons towards the ventral bulb [59].

### 3.3. Anteroposterior mapping

#### 3.3.1. The role of ORs

Unlike the dorsoventral and mediolateral axes, the anteroposterior position of glomeruli in the OB is independent of the spatial position of their source OSNs in the OE [7]. Some genetic manipulations, such as double knockout of the axon guidance molecules ephrinA5 and ephrinA3 [60], or deletion of the hyperpolarization-activated ion channel subunit HCN1 [61], have been associated with selective posteriorization of glomerular positions, but the mechanisms underlying these axis-specific effects remain obscure. By contrast, the development of the anteroposterior axis is known to have an important contribution from another, distinct information source: OR expression. OR expression patterns in the OE are independent of OB-derived signals [62]; however, that OR identity can be a determinant in glomerular position was suggested by the fact that ectopically expressed ORs can lead to normal glomerular formation [63–66], and that even a change in the level of OR expression can produce a shift in glomerular location [67]. Knocking out individual ORs would seem like the most direct strategy to assess their contribution to glomerular development, but in these cases mechanisms of OR choice led to knock-out OSNs expressing a range of replacement ORs and, as a population, targeting a broad array of glomeruli [66,68]. Instead, a series of ingenious OR substitution experiments replaced endogenous host OR genes with specific alternative donor genes. These studies found that OR identity was entirely sufficient to determine glomerular position when the host and donor like-OSNs usually mapped to near-neighbour glomeruli, and was a contributing factor when host and donor glomeruli were usually distantly positioned [13,69,70]. Importantly, the positional shifts of glomeruli in these distant OR substitution experiments were strongest in the anteroposterior axis [70]. Given the temporal variability in onset of OR expression across different like-OSN populations [71], perhaps the anteroposterior gradient in the timing of glomerular development [36] could be contributed to, at least in part, by the developmental dynamics of OR choice. As is the case for coarse targeting in the dorsoventral axis (see §3.2), temporal factors in maturation are a potentially crucial determinant of differences in targeting for distinct like-OSN subtypes.

#### 3.3.2. G-protein G_s_

How can OR identity determine glomerular position? One hypothesis was that it does so via odorant-driven activity in OSNs; however, knockouts for key components in the OR-dependent olfactory transduction cascade, such as G_olf_ [72] and CNGA2 [73,74] showed no deficits in coarse nose-to-brain mapping. By contrast, knocking out a different node of the canonical transduction cascade, the cAMP-synthesizing enzyme AC3, resulted in highly disrupted OSN-to-OB projections [64,75,76]. Might there be a distinct pathway, separate from odorant-driven activity, by which ORs can determine AC3 activity? Indeed, immature OSNs express the AC3-activating G-protein G_s_, the ectopic expression of which results in glomerular coalescence [64]. In addition, non-OR receptors which couple to G_s_ can substitute for ORs in determining OSN identity and glomerular formation [64,67]. Moreover, OSNs expressing mutant ORs which cannot interact with G_s_ are unable to form glomeruli, a phenotype which can be rescued by concomitant expression of constitutively active G_s_ [77], while targeted conditional G_s_ deletion leads to the rI7 glomerulus forming erroneously in the anterior OB [78]. Finally, ectopic non-odorant binding receptors with a low level of associated intrinsic G_s_ activity produce an anterior shift in glomerular position, while the same receptors coupled to high-activity G_s_ produce a posterior shift in glomerular position [78]. This all suggests that agonist-independent G_s_ activity levels, determined by OR expression in immature OSNs, are crucial contributors to the anteroposterior position of developing glomeruli.

#### 3.3.3. cAMP/PKA

The key roles for G_s_ and AC3 in glomerular formation, and specifically in anteroposterior positioning, suggest that this process may depend upon levels of cAMP in OSNs. This non-canonical OR-dependent pathway could in fact function locally, in presumptive OB glomeruli, since ORs are translated and expressed in OSN axons [67,79,80] (figure 1), and focal OR stimulation can produce cAMP increases in OSN terminals [81]. Indeed, an elegant series of genetic manipulations showed that altering activity of the cAMP-activated enzyme PKA produced bidirectional shifts in the position of the ectopic rI7 glomerulus, with low PKA activity relocating the glomerulus anteriorly, and constitutively high PKA activity pushing the glomerular position more posterior [77] However, this G_s_-cAMP-driven contribution appears to be non-universal across different like-OSN populations since manipulations of this pathway did not produce similar anteroposterior shifts in the position of the M71 glomerulus [82].

#### 3.3.4. Nrp1

The mechanisms by which cAMP signalling might regulate anteroposterior glomerular positioning are similarly contested. The axon guidance molecule Nrp1 has a cAMP-dependent expression pattern with a coarse low anterior to high posterior overall gradient in the OB [75,77,78], and its expression levels can be controlled by changes in agonist-independent G_s_ activity [78]. Raising or depleting Nrp1 levels with OSN-specific manipulations led to posterior and anterior shifts, respectively, in rI7 glomerular position [83]. However, these rI7 effects could not be independently replicated, nor did conditional OSN Nrp1 knockout produce consistent changes in the anteroposterior positioning of a different glomerulus, M71 [84]. The role of Nrp1 in anteroposterior glomerular mapping is therefore currently unclear.

#### 3.3.5. Sema3A

A repulsive ligand for Nrp1, Sema3A has also been implicated in anteroposterior glomerular positioning. Specific deletion of Sema3A in OSNs leads to disruption in the spatial order of axons within pre-OB olfactory nerve bundles, and also produces anterior shifts in glomerular location [83]. In addition, anteriorly relocated glomeruli are observed when the interaction between Nrp1 and Sema3A is constitutively perturbed [85]. However, those manipulations are also associated with dorsal glomerular repositioning [85], while full (not just OSN-specific) Sema3A knockout produces complex glomerular relocation phenotypes across multiple axes [86–88]. This suggests that OSN-based Nrp1-Sema3A signalling might be involved in anteroposterior glomerular patterning, but also that the same pathway and/or OB-expressed Sema3A could have additional roles in coarse nose-to-brain mapping [28].

#### 3.3.6. Local OR ligands

Because ORs are present on OSN axon terminals in the OB, there is the possibility that their activation of downstream signalling pathways might not be solely agonist-independent [78] but might also be controlled by the presence of OR-binding ligand molecules within the brain. This could provide an alternative means for OR identity to control (anteroposterior) glomerular positioning. The first such OR ligand to be identified is PEBP1, which is expressed in OB juxtaglomerular neurons in a patchy distribution with an overall high-anterior to low-posterior gradient, and which can produce calcium transients when applied to the terminals of OSNs expressing specific ORs [89]. Knockout of PEBP1, however, produced only small and rather variable shifts in the position of the P2 glomerulus, whose OSN axons were able to respond to the ligand. It remains to be seen whether PEBP1 interacts with other potential OR ligands, as well as ligand-independent OR-driven signalling, in a combinatorial code for establishing OB glomerular location.

## 4. Local glomerular segregation and coalescence of OSN inputs to the OB

Once OSN axons have used all of the above mechanisms to arrive at roughly the right three-dimensional position in the developing OB, their next task is to sort themselves locally to form coalesced glomeruli of like-OSN axons that are segregated from similarly coalesced glomeruli of neighbouring like-OSNs (figure 2). This process appears to be dependent only upon the OSN axons themselves because it can happen in the absence of their usual postsynaptic partner cells. A series of classic studies showed that OSNs forced to regenerate by the removal of part or all of the OB can form anatomically ‘glomerular-like’ structures in deeper layers of the OB that they would never target [90], in non-OB forebrain tissue that fills the space left by the missing OB [91,92], or even in tissue from developing occipital cortex transplanted into the space where the OB once was [93]. Subsequent studies using genetically labelled like-OSNs found that the P2 glomerulus could form normally in mice lacking different populations of postsynaptic target neurons—in either Tbr1 knockouts that have no mitral/tufted cells, or Dlx1 + 2 knockouts that lack most bulbar GABAergic interneurons [42]. The P2 glomerulus can even form in Gli3 knockout mice that lack an OB entirely and instead have an amorphous fibrocellular mass in its place [94]. This wholly OSN-based local segregation and coalescence process is now known to be driven by attraction and repulsion mediated by a combination of different cell surface molecules, at least partly under the control of neuronal activity.

### 4.1. Kirrel2/3 and ephrin-A5/EphA5

The cell surface molecules kirrel2 and kirrel3 have recently been shown to have structures that can homo- but not heterodimerize [95], allowing them to mediate selective homophilic axonal adhesion. By contrast, the classic axon guidance molecules ephrin-A5 and EphA5 mediate repulsive axonal interactions. All four of these cell surface molecules have variable expression across developing OB glomeruli, with inverse correlations between levels of kirrel2 and kirrel3, and between EphA5 and ephrin-A5 [96]. In some elegant mosaic expression experiments, a subset of like-OSNs with high expression of kirrel2 or kirrel3 was found to segregate from like-OSNs that expressed the same OR but which had no kirrel2 or kirrel3 expression [96]. This effect indicates that OR proteins themselves are unlikely to directly drive glomerular convergence, despite their expression on OSN axon terminals [67,79,80]. It also suggests that differences in kirrel expression between neighbouring OSNs can be sufficient—via homophilic interactions, and with an additional potential contribution of ephrinA5/EphA5-mediated repulsion—to drive them to form segregated glomeruli [96]. Subsequent investigation of kirrel2 or kirrel2 + 3 knockout mice also found glomerular segregation deficits which were dependent upon the particular like-OSN population studied [97]. These effects were not linearly associated with levels of kirrel expression, however [97], suggesting that other cell surface molecules might also contribute to local glomerular segregation in a combinatorial and/or redundant manner.

### 4.2. BIG-2 and Pcdhs

Indeed, other such molecules have now been identified. They include BIG-2, which displays a mosaic pattern of glomerular expression levels distinct from those of kirrel2 or ephrin-A5, and the knockout of which produces multiple glomerular innervation in ectopic locations [98]. The Pcdh family also contributes significantly to glomerular coalescence and segregation. Pcdh*α* mutant mice have no deficits in coarse mapping to the correct OB position, but their like-OSN populations form multiple small extraneous glomeruli [99]. Even more severe deficits in glomerular segregation are seen when levels of different Pcdh clusters—usually expressed in diverse combinations in different populations of like-OSNs—are simultaneously either reduced or increased [100]. Pcdh10 also has variable levels of expression from glomerulus to glomerulus, and its mis-expression produces a lack of glomerular convergence [101]. Together, the effects of manipulating these different cell surface molecules suggest there is a combinatorial code for glomerular segregation/coalescence, whereby different levels of expression of a variety of different molecules mediating homophilic adhesion or heterophilic repulsion led to reliable spatial organization of like-OSN axon populations. Indeed, staining for multiple cell surface proteins can reveal a beautiful glomerular identity map [102,103]. What is currently unclear is exactly how many molecules contribute to, or are essential for, every glomerulus to coalesce successfully, and how their actions interact at the intracellular level.

### 4.3. Dependence on neuronal activity

What controls the differential expression levels of cell surface molecules involved in local glomerular segregation and coalescence? One key factor is neuronal activity. Manipulations of OSN activity levels, often via sensory deprivation with unilateral naris occlusion (UNO) or via mosaic knockout of the CNG channel that underlies odour-evoked OSN firing, have been shown to alter levels of expression of kirrel2/3, ephrin-A5/EphA5 [96], BIG-2 [98], Pcdh10 [101,103] and Sema7A [103]. These effects are often in different directions for differentially acting molecules—for example, UNO is coupled with increased expression of kirrel2 and EphA5, but decreased expression of kirrel3 and ephrin-A5 [96]. Differential odorant-driven activity across different populations of like-OSNs may therefore drive differential cell surface molecule expression and therefore glomerular segregation and coalescence.

### 4.4. Dependence on spontaneous activity

However, although this activity-dependent expression has been most thoroughly investigated using manipulations of activity driven by sensory experience, recent work suggests there may also be a strong role for spontaneous OSN firing in cell surface molecule expression. Mature OSNs can fire action potentials spontaneously, at rates and patterns that depend on the OR they express [104,105]. More relevant for developmental processes, OSNs in acute slices from the neonatal OE show spontaneous calcium transients that have no spatial structure, but are differentially temporally patterned between different populations of like-OSNs [103]. Spontaneous OSN firing is not only present and distinct between OSNs expressing different ORs, but it is also necessary for glomerular segregation. Multiple studies have shown that in mice with OSNs that constitutively overexpress the inward-rectifying potassium channel Kir2.1, spontaneous activity is specifically decreased while odour-evoked OSN firing remains normal [106,107]. These mice show severe deficits in glomerular coalescence, with disordered, diffuse projections of like-OSN axons over wide areas of the OB's glomerular layer [106–108]. Moreover, a recent study has now demonstrated that temporal patterns of spontaneous activity in developing OSNs are instructive in determining expression levels of cell surface molecules crucial for glomerular segregation/coalescence. Using specifically patterned optogenetic manipulations of OSN activity inspired by different spontaneous firing observed in distinct like-OSN populations, levels of kirrel2, Sema7A and Pcdh10 expression can be precisely controlled [103]. In addition, elevating overall OSN activity using optogenetic stimulation was sufficient to induce glomerular segregation of stimulated versus unstimulated like-OSN sub-populations [103]. However, to truly demonstrate an instructive mechanism it remains to be shown that such segregation can be achieved by the precise control of firing patterns at the same overall rate of activity. It will be exciting to follow future work aimed at understanding how spontaneous activity patterns can control specific gene expression in developing OSNs, and whether there is sufficient difference in spontaneous activity patterns across different like-OSN populations to entirely code for the whole cell surface molecule repertoire needed for glomerular segregation and coalescence.

## 5. Refinement of erroneous projections in OSN inputs to the OB

The final stage in the development of OSN inputs to the OB is the removal of stray projections to erroneous targets which persist after the process of local glomerular segregation and coalescence is largely complete (figure 2) [11,45]. Again, there is a role for neuronal activity here, although in this late stage of OSN-to-OB maturation it is primarily sensory experience-driven OSN activity which appears to be most important.

### 5.1. The role of sensory experience

A crucial consideration in the later refinement of OSN-to-OB projections is that different like-OSN populations, and their respective glomeruli, can develop at very different rates [74,76]. This may at least partially explain why the first studies investigating mice deficient in key components of the olfactory transduction cascade—which assessed the early-developing P2 glomerulus only—reported no effects at all on glomerular targeting [72–74]. By contrast, labelling M72-expressing like-OSNs, whose projection develops much later, revealed elevated numbers of multiple M72-innervated glomeruli in postnatal CNGC knockout mice that lack odorant-evoked activity [74,109]. Subsequent investigations used early postnatal UNO to reduce sensory-evoked activity in OSNs, and observed the retention of supernumerary glomeruli in ectopically projecting like-OSN populations [110] or in OSN axons expressing particular combinations of hybrid ORs [69]. In a landmark study, early postnatal UNO was associated with the persistence of multiple glomeruli in both the late-developing M71 and M72 like-OSN populations; moreover, sensory deprivation ceased to be effective if started past different critical time points for each of these populations: ∼P15 for M72-expressing OSNs, but ∼P25 for the even later-developing M71-expressing OSNs [11]. Local glomerular refinement is also disrupted in OMP knockouts which have abnormal odorant response properties [111]. By contrast, learning an odorant aversion task was associated with more rapid glomerular refinement in an OSN population responsive to the learnt stimulus [112]. The development of some (later-developing) OSN types, therefore, has the potential to be highly regulated by postnatal sensory experience. Importantly, the supernumerary glomeruli retained in UNO mice were ‘heterogeneous’ in that they contained axons from OSNs expressing different ORs, while in all animals there was always at least one ‘homogeneous’ glomerulus in which all OSN axons appeared to express the same labelled OR [11]. This suggests that the experience-dependent process of refinement involves the removal of stray projections that erroneously terminate in glomeruli dominated by axons from other like-OSN populations, while retaining the projections that form a homogeneous segregated termination zone. How might such a refinement process be mediated?

### 5.2. The role of competition

Multiple lines of evidence support the idea that OSN axons compete for space in the developing glomerular layer, with the subsequent removal of projections that are unable to join enough of their like-OSN counterparts. For example, the maintenance of a glomerulus by OSNs expressing a transgenic OR depends on the number of OSNs expressing that OR [113], and transgenic OR-expressing OSNs are only able to segregate from axons expressing the endogenous version of the same OR when they are of sufficient numbers [46]. Mosaic knockout of CNGA2 also produces a sensory experience-dependent preferential loss of non-odour-driven OSNs, versus their wild-type sensory-driven neighbours, over developmental time [114]. In addition, manipulations of OSN function are associated with more severe glomerular targeting phenotypes when they occur sparsely (i.e. on a background where the majority of OSN axons function normally) than when they occur across all OSNs. For example, P2 axons have severe targeting deficits when only they, but not other OSNs, have decreased spontaneous activity because of Kir2.1 overexpression. Moreover, blocking transmitter release from OSN terminals has no effect on glomerular convergence if carried out by tetanus toxin expression in all OSNs, but produces stray projections and rapid cell death if expressed in isolated neurons [106].

### 5.3. Cellular and molecular mechanisms

The fact that individual developing OSN axons have never been observed to project to multiple glomeruli [48] suggests that the process of refining erroneous nose-to-brain projections occurs at the whole-OSN level, rather than at the level of individual axon branches. Indeed, retrograde signalling from axon terminals in the OB to the soma in the OE is possible—odorant or cGMP-analogue-based stimulation in the OB can induce increased phosphorylated CREB signal in OSNs in the OE [115]—so local axonal conditions are capable of inducing whole-cell signalling events. The most parsimonious explanation for the above effects of activity-dependent competition is that erroneously projecting OSNs undergo rapid cell death, although direct evidence for this has not yet been obtained [11,47]. A mechanism consistent with the data so far would involve a winner-take all competition for glomerular space, based on coincident activity among like-OSN axons. In this model, a threshold number of co-projecting like-OSNs in a given glomerular region would be able to out-compete any other OSN axons for limited survival-determining resources. Crucially, this competition would be based on the coincident timing of activity correlated most strongly among OSNs expressing the same OR, because any model based purely on overall *rates* of activity would never permit the establishment of glomeruli by OSNs with low activity rates and/or stimulus-induced inhibition [116–118].

What might be competed for? The trophic factor BDNF appears to play no role in the initial targeting of OSNs to the OB [119] but ectopic glomeruli are found in mice deficient in its receptor p75(NTR) [120]. Furthermore, BDNF signalling is required for the neurotransmitter release-dependent maintenance of branching structure of OSN axons within maturing glomeruli [47]. BDNF is not expressed in OSNs, being found instead in mitral/tufted cells (M/TCs) and juxtaglomerular cells in the OB [47], suggesting that any role it might play in activity-dependent OSN survival would occur via synaptic interactions between OSNs and their postsynaptic targets. Indeed, these synapses are present, functional and capable of plasticity during the early postnatal period of glomerular refinement [121,122], and NMDA receptor activity, like BDNF signalling, is needed to maintain within-glomerular axon branching in this time period [47]. However, if the ‘glomerular-like’ structures that form when OSNs are forced to project to ectopic, non-OB targets [94] are fully refined and homogeneous—an important and entirely open question—this would indicate that interactions with postsynaptic targets are not required for glomerular refinement. Then, if BDNF and/or activity-dependent cell survival are required for this refinement process, it will be fascinating to learn how OSN axons could independently—without synaptic interactions—compete for resources that they themselves do not produce. Of course, it is also possible that the key mechanism(s) might actually be provided by another signalling pathway altogether. There is much to address here in future work!

## 6. Maintaining and repairing the nose-to-brain map

We have reviewed in detail the processes and the developmental patterns driving the establishment of glomerular targeting by OSNs, but how is the nose-to-brain map maintained in the adult brain? Olfactory sensory neurons are highly vulnerable to insult via injury, infection, inflammation or toxin exposure, and so undergo continual production throughout life via postnatal and adult neurogenesis [123]. This means that the highly organized chemospatial glomerular map established during development must be maintained while its constituent cells, and their OB-projecting axons, are continually replaced. Under constitutive, baseline conditions this map maintenance is rather successful, with only a few glomerular targeting errors occurring in later adult life [124]. Newly adult-generated immature OSNs can make functional synapses with OB cells to provide downstream circuits with specialized olfactory information [49,125], and this suggests that activity (and possibly synapse-dependent processes) might determine their glomerular targeting. Indeed, expressing tetanus toxin to block glutamate release from OSNs starting from postnatal day (P)21 produces diffuse axonal projections [106], while lowering spontaneous activity via Kir2.1 overexpression from P21 [106] or P30 [107] also leads to a loss of glomerular convergence and the emergence supernumerary glomeruli around after around a month. The processes of constitutive map maintenance appear entirely different from those that set up the glomerular projection in the first place, since switching off Kir2.1 expression past P5—or reversing other, non-activity-dependent developmental manipulations at the same time point—does not allow recovery of an induced multiple-glomerular phenotype, even when these manipulations are specific to one population of like-OSNs on an otherwise normal background [108]. A similar multiple glomerular effect produced by genetic OR mis-expression is also only evident if induced constitutively or in the early postnatal time period [126]. These studies suggest that the glomerular map is maintained by newly generated OSNs following cues provided by their pre-existing like-OSN counterparts. If these older OSNs are missing, the map cannot be successfully re-established.

This is demonstrated clearly in studies of nose-to-brain regeneration, where OSNs are capable of wholescale reconstitution following widespread OE cell death [127]. However, the accuracy of reconnected OSN-to-OB mapping is dependent upon the severity of the initial degeneration. Selectively ablating 95% of just P2-expressing OSNs results in accurate re-growth of new P2 axons to the correct, discrete glomerular location [128], while mild detergent treatment of the OE allows accurate regeneration at the level of certain glomerular groups [129]. The specific olfactotoxin methimazole allows post-degeneration recovery of glomerular position with a few supernumerary projections [130]. These effects are mirrored by a similar but stronger phenotype after more extensive OE disruption with another olfactotoxin, dichlobenil [131], and a similar fragmented functional glomerular activation pattern after OE lesion with inhaled methyl bromide [132]. The most severe disruption occurs with injury to the olfactory nerve, complete transection of which produces disrupted areal patterning of the OB [133] and significant errors in like-OSN projections [134]. New OSNs seem to need mapping cues provided by at least some remaining like-OSNs (or their remnants) in order to re-form an accurate nose-to-brain projection.

## 7. Conclusion

We currently understand many of the basic processes involved in establishing the complexity and precision of the nose-to-brain map of odour identity. This complexity is represented not only in the many interacting factors shaping this process, but also in the differences in timing and developmental mechanisms that occur between different parts of the OB, and between OSNs expressing different ORs. Nevertheless, a clear picture is building of how genetically determined and activity-dependent processes interact to produce a highly precise OSN-to-OB projection. Some critical steps in the development and maintenance of this projection still remain unclear, however. How do individual OSN axons change over time as glomerular coalescence and refinement occur? What do OSNs compete for when glomerular space is apportioned? And how do the axons of newly generated OSNs find their path to the correct glomeruli in the adult brain? Answering these open questions, along with the many others outlined above, will be key to advancing our knowledge of nose-to-brain map formation in the years to come.

## Data Availability

This article has no additional data.
